# The thermodynamic brain

**DOI:** 10.1186/s13054-014-0693-8

**Published:** 2014-12-12

**Authors:** Joseph Donnelly, Marek Czosnyka

**Affiliations:** Division of Neurosurgery, Department of Clinical Neurosciences, Addenbrooke’s Hospital, University of Cambridge, Hills Road, Cambridge, CB2 0QQ UK; Institute of Electronic Systems, Warsaw University of Technology, Nowowiejska 15/19, Warsaw, Poland

## Abstract

Apart from its complex functionality, the brain is a robust thermodynamic machine; the tissue metabolic rate is high and it is thermally shielded by a skull. Therefore, if there is no high-volume blood flow to cool and stabilize the brain temperature, the possibility of unstable behavior seems to be high. Inflowing arterial blood is normally cooler than the brain tissue temperature, and outflowing venous blood is normally warmer than arterial blood but cooler than the brain tissue. Brain blood flow can thus be understood as a cooler for the brain. Pros and cons of clinical measurement, with clear indication for a multimodal monitoring approach, are discussed along with a brief review of basic facts known about temperature, cerebral blood flow and volume, intracranial pressure, and compartmental compliances of the brain.

A large quantity of experimental works, sometimes with conflicting conclusions, have been published about the regulation of core temperature and brain temperature. Bedside clinical studies, however, are not that common. Measurement of cerebral blood flow in comparison with core body temperature in traumatic brain injury (TBI) patients, as presented in a recent issue of *Critical Care* by Stretti and colleagues, is therefore worth commendation [1].

Cooling the body to protect the brain and preserve life may sound like something from science fiction. However, cooling seems to have very real beneficial effects. Some of our friends in the animal kingdom can withstand remarkable physiologic insults, at least when they are cold. For example, *Spermophilus tridecemlineatus* (ground squirrels) can tolerate 90% reductions in cerebral perfusion without any neurologic deficit, provided their temperature is reduced to 10°C during hibernation [2]. In fact, cooling has been used as a therapy in medicine for decades [3]. Since its inception, enthusiasm for cooling has waxed and waned in the domains of cardiopulmonary bypass, cardiac arrest, stroke, and TBI [4]. Because temperature has the potential to alter cerebral metabolism, blood flow and intracranial pressure (ICP), therapeutic cooling has been proffered as a management strategy after TBI. However, clinical trials in TBI have not been conclusive to date [5,6], prompting a new large-scale multicenter European trial [7]. In any case, clinical trials usually differ from scientific procedures because they provide pragmatic answers for clinicians (if conclusive) but often not for scientists. The need to establish a link between clinical utility and scientific rationale forces us to return to simple questions such as that posed by Stretti and colleagues: what are the brain hemodynamic effects of temperature changes?

The basic tenants underpinning therapeutic cooling in TBI are related to the fundamental relationship between temperature and the rate of biochemical reactions common to all species [8], and the effect of temperature on ICP. The brain may be particularly sensitive to changes in temperature for two reasons: the brain is highly metabolically active; and, due to the rigid cranium, temperature-induced changes in metabolism and cerebral blood volume can result in changes in ICP. The ICP itself is governed by the volume of the various compartments in the skull; namely, the vascular, parenchymal, and cerebrospinal fluid (CSF) compartments. The question then arises as to which component of ICP temperature affects.

The vascular component is the obvious choice because decreasing temperature increases vascular tone in the small pial vessels [9], and perhaps even in the basal arteries [10]. Aside from altering the vascular component of ICP, it is also possible (and as yet unknown) that the CSF or parenchymal compartments are altered – for example, by altering CSF production or reabsorption, or by affecting the osmotic composition of the parenchymal interstitium. Separating such components will be difficult to achieve experimentally and especially clinically.

Stretti and colleagues used transcranial Doppler (TCD) ultrasonography during alteration in body temperature after TBI in an attempt to further our understanding of the cerebral hemodynamic consequences of cooling. TCD is a stethoscope for the brain, and its bedside use in several scenarios should be more widespread. The advantages of TCD measurements on arrival at neurocritical care, to make a quick assessment and decide about the first few hours’ management strategy, have been highlighted recently [11]. TCD measures blood flow velocity, not volumetric flow, and because it has a pulsatile component can (and should) be analyzed with pulse waveform signal processing methodology. In addition, combining TCD velocity measures with arterial blood pressure and cerebral perfusion pressure can provide further insight into cerebral hemodynamics by describing the autoregulation, vascular compliance, resistance, time constant, wall tension, critical closing pressure, and other parameters.

In the current study, the relationships between core body temperature and cerebral hemodynamics were studied in two groups of TBI patients: those with a fever who were subsequently cooled (defervescence group); and those who were hypothermic who were warmed to normothermia (rewarming group) [1]. The mean flow velocity observed in the rewarming group is nearly twice lower than that in the defervescence group. This disproportion remains apparent even if the upper temperature in rewarmed patients was close to the lower temperature in the defervescence group. This observation may suggest that there is no one universal temperature–cerebral blood flow relationship, and other physiological variables obviously play a role. The authors report that with lower temperature we see lower mean arterial pressure and lower TCD pulsatility index. This is a novel finding. The pulsatility index is theoretically proportional to the pulsation of blood pressure and (nonlinearly) to a product of cerebrovascular resistance and arterial compartmental compliance multiplied by the heart rate, but inversely proportional to cerebral perfusion pressure [12]. We still know little about vascular resistance and compliance when temperature varies, and cerebral perfusion pressure is reported to stay constant at least in the defervescence group; therefore, it is possible that the lowering of arterial blood pressure pulse amplitude is responsible for the reduced pulsatility index with lower temperature.

In conclusion, Stretti and colleagues touch complex and still poorly chartered phenomena. Because of the multifactorial interactions between brain injury, temperature control, cerebral blood flow, and autoregulation, the answer to all questions is impossible within an observational study design based on a limited number of cases. Nevertheless, this paper opens a thought-provoking discussion and, we hope, will stimulate further clinical research in the area of the thermodynamic brain.

## Abbreviations

CSF: Cerebrospinal fluid
ICP: Intracranial pressure
TBI: Traumatic brain injury
TCD: Transcranial Doppler
